# RD3 loss dictates high-risk aggressive neuroblastoma and poor clinical outcomes

**DOI:** 10.18632/oncotarget.5204

**Published:** 2015-09-08

**Authors:** Faizan H. Khan, Vijayabaskar Pandian, Satish Kumar Ramraj, Sheeja Aravindan, Mohan Natarajan, Seifollah Azadi, Terence S. Herman, Natarajan Aravindan

**Affiliations:** ^1^ Department of Radiation Oncology, University of Oklahoma Health Sciences Center, Oklahoma City, OK, USA; ^2^ Stephenson Cancer Center, Oklahoma City, OK, USA; ^3^ Department of Pathology, University of Texas Health Sciences Center at San Antonio, San Antonio, TX, USA; ^4^ Department of Cell Biology, University of Oklahoma Health Sciences Center, Oklahoma City, OK, USA

**Keywords:** RD3, neuroblastoma, tumor suppressor, high-risk aggressive neuroblastoma, metastasis

## Abstract

Clinical outcomes for high-risk neuroblastoma patients remains poor, with only 40–50% 5-Year overall survival (OS) and <10% long-term survival. The ongoing acquisition of genetic/molecular rearrangements in undifferentiated neural crest cells may endorse neuroblastoma progression. This study recognized the loss of Retinal Degeneration protein 3, RD3 in aggressive neuroblastoma, and identified its influence in better clinical outcomes and defined its novel metastasis suppressor function. The results showed ubiquitous expression of RD3 in healthy tissues, complete-loss and significant TNM-stage association of RD3 in clinical samples. RD3-loss was intrinsically associated with reduced OS, abridged relapse-free survival, aggressive stage etc., in neuroblastoma patient cohorts. RD3 was transcriptionally and translationally regulated in metastatic site-derived aggressive (MSDAC) cells (regardless of CSC status) *ex vivo* and in tumor manifolds from metastatic sites in reproducible aggressive disease models *in vivo*. Re-expressing RD3 in MSDACs reverted their metastatic potential both *in vitro* and *in vivo*. Conversely muting RD3 in neuroblastoma cells not only heightened invasion/migration but also dictated aggressive disease with metastasis. These results demonstrate the loss of RD3 in high-risk neuroblastoma, its novel, thus-far unrecognized metastasis suppressor function and further imply that RD3-loss may directly relate to tumor aggressiveness and poor clinical outcomes.

## INTRODUCTION

Neuroblastoma (NB), a predominant tumor of early childhood [1, 2], accounts for 9.1% of pediatric cancer deaths [3–5]. Though overall survival (OS) rates have significantly increased over the last three decades [3, 5], the OS rates mask the significant variability in outcomes for different risk groups. Children who present with favorable NB (comprising about 40% of total NB patients) show a complete cure through spontaneous regression or spontaneous maturation. However, more than half of the patients with high-risk aggressive NB will relapse with hematogenous metastasis [6] despite intensive multimodal therapy, which may include chemotherapy, surgery, external beam radiotherapy, myeloablative chemotherapy with autologous stem cell transplant, and/or differentiation therapy with 13-cis-retinoic acid [3, 5, 7–14]. Given the disease's heterogeneity, resistance, and poor hematological reserve, the likelihood of a cure after relapse of high-risk disease is significantly less, with 5-year OS of 40–50% compared with the >95% OS with low and 90–95% OS with intermediate-risk disease (http://www.cancer.org/cancer/neuroblastoma/detailedguide/neuroblastoma-survival-rates). However, it has been appropriately suggested that these single numbers can be misleading because of the extremely heterogeneous prognosis based on the neuroblastoma patient's age, stage, and biology (http://www.cancer.gov/types/neuroblastoma/hp/neuroblastoma-treatment-pdq/#link/_866). To that note, approximately 70% of patients with neuroblastoma have metastatic disease at diagnosis. Individual Children's Oncology Group (COG) studies have shown that long term survival rates were poor for patients with stage 4 disease, with only 2% ten-year OS compared with the 38–71% OS for those with low-risk disease [13, 15]. A relapse timeline of less than 18 months for the first recurrence and only 8.7 and 3.8 months for second and third recurrences [9, 10] suggests that molecular rearrangements could drive ongoing acquisition of chemo- and radiation-resistance and pro-oncogenic adaptations in aggressive NB. Identifying the crucial molecular targets, defining their orchestration, and understanding the signal transduction flow-through that switches favorable NB to aggressive high-risk NB could lead to the development of an efficient and improved targeted therapeutic strategy and better patient outcomes.

The retinal degeneration protein 3 (RD3/LCA12/C1orf36) gene encodes a 195 amino-acid long protein with a relatively low molecular mass (22kDa); RD3 includes putative coil-coil domains at amino acids 22–54 and 115–141 and several conserved sites for protein modification [16]. Retinal degeneration studies have shown that genetic defects in the RD3 gene and subsequent mutation (homozygous c.319 C → T in exon 3) generates a stop codon, thereby producing a less stable truncated protein [17]. Recently, a number of cutting-edge studies underscored the importance of RD3 in photoreceptor cell survival, and provided insights into the function of RD3 in photoreceptor cells and the mechanism by which RD3 mutations cause photoreceptor degeneration (Supplementary Figure S1) [16, 18, 19]. RD3 binds to guanylate cyclases GC1 and GC2, translocates GCs from the ER to the photoreceptor outer segment, and suppresses the basal enzymatic activity of GCs [16, 18, 19]. In addition, *RD3* mice lack GC expression in the retina. These findings highlight the importance of RD3 in maintaining GC expression and stability [16]. Though RD3 has been shown to co-localize with the tumor suppressor promyelocytic leukemia (PML) protein [17], the functional role of this crucial protein in cancer cell biology (or in any other disease systems) has been thus far overlooked. The results presented here show the significant loss of RD3 in high-risk aggressive metastatic neuroblastoma *in vivo, ex vivo*, and in clinical tumor specimens. The data underscore the potential role of RD3 in the switch from favorable neuroblastoma to the high-risk aggressive disease.

## RESULTS

### RD3 is constitutively expressed in normal tissues

**Figure 1:**
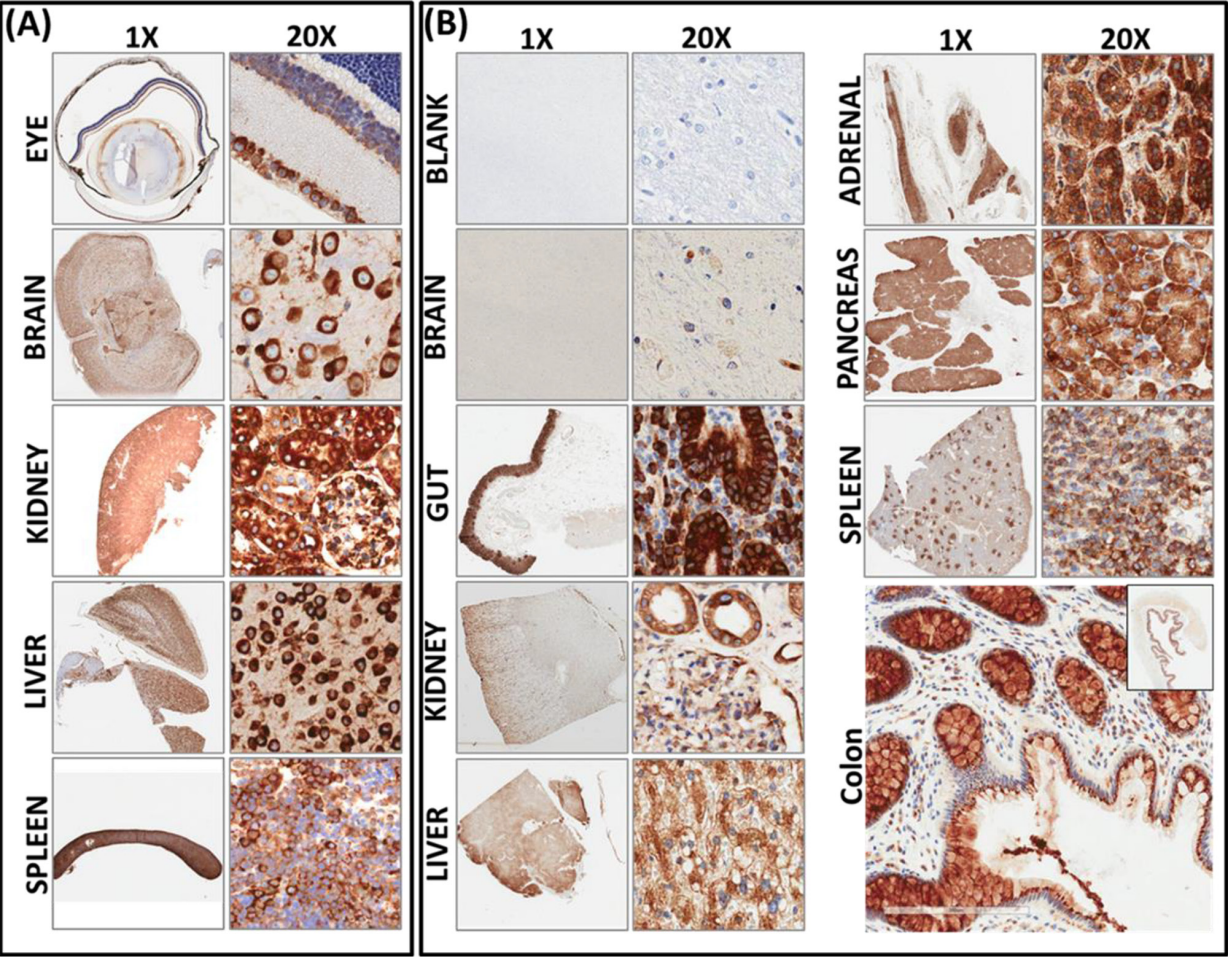
Constitutive expression and localization of RD3 in normal mouse and human tissues **A.** Representative photomicrographs (1x and 20X) showing RD3 localization and expression in healthy mouse tissues. Tissue macroarray constructed with healthy mouse eye, brain, kidney, liver and spleen tissues coupled with automated IHC showing strong positivity of RD3 expression localized in perinuclear, cytoplasmic, and nuclear regions. **B.** RD3 localization and expression in normal human tissues. Corresponding section with no-primary antibody IHC(s) serves as the negative control. RD3 is ubiquitously expressed in in normal human brain, gut, kidney liver, pancreas, adrenal gland, spleen and colon tissues and highly localized in perinuclear regions, with some positivity in nuclear and cytoplasmic regions. Enlarged photomicrograph of colon depicts RD3 cellular localization.

To better underscore the significance of RD3 loss in high-risk neuroblastoma, first, we investigated the expression levels of RD3 in normal mouse and human tissues. Apart from retinal RD3, its expression is poorly understood in any tissues in any mammalian systems. To examine RD3 cellular localization and expression, we studied a customized tissue macro-array comprising mouse brain, kidney, liver, and spleen (Figure 1A). RD3 IHC revealed strong positivity in all tissues analyzed. Positive RD3 staining appeared in brown and was predominantly localized in the perinuclear area (see 20X panel, Figure 1A). We also observed nuclear and cytoplasmic localization of RD3. Conversely, no-primary negative controls revealed no staining (data not shown). Aperio quantification analysis revealed a high positivity score in mouse brain (35.119 ± 4.511), kidney (92.563 ± 7.641), liver (42.367 + 3.303), and spleen (78.748 + 3.037). Kidney exhibited relatively higher positivity for RD3 staining. Parallel staining of mouse eyes demonstrated a strong RD3 positivity in the inner retinal membrane and served as the positive controls. Together, these results show the constitutive expression of RD3 in normal mouse tissues.

To further substantiate our findings for translation to clinical settings, we examined the localization and expression of RD3 in normal human tissues (Figure 1B). Sections of normal control human tissues, including brain, gut, kidney, liver, adrenal gland, pancreas, and spleen, were a kind gift from Dr. Kar-Ming Fung, Professor and Director of the Stephenson Cancer Center - Cancer Tissue Pathology Core. Brain samples labelled with no-primary antibody served as the negative controls. We observed a strong positivity in all tissues analyzed. Visually, we found strong positivity in the human gut and in the pockets of adrenal tissues. Aperio image analysis and quantification analysis revealed high positivity in human kidney (63.995 ± 2.140), colon (65.821 ± 3.74), pancreas (94.902 ± 7.429), spleen (78.489 ± 12.98), liver (91.142 ± 6.059), adrenal gland (40.827 ± 4.602), and brain (2.28 ± 0.182). Taken together, these results demonstrate that RD3 is localized in perinuclear, nuclear, and cytoplasmic regions, and is highly expressed in normal mouse and human tissues at basal conditions.

### Regulated RD3 transcriptional machinery in high-risk disease

For this, we examined the levels of RD3 transcription between non-metastatic and aggressive metastatic neuroblastoma cells in both *in vivo* and *ex vivo* settings utilizing the characterized mouse model of high-risk aggressive and metastatic neuroblastoma [20–22]. In brief, sub-cutaneous injection of (5 × 10^6^) human SH-SY5Y cells suspended in Matrigel resulted in the development of xenografts (~200 mm^3^) over a period of 30 days at least in ~70% of the animals. However, about 30% of the mice that received identical clones under similar conditions were presented with multiple clinically-mimicking metastatic tumors in the mediastinum and retroperitoneal, pelvic, abdominal, and chest cavities over an extended period of ~50–60 days. Development of this aggressive disease with metastatic dissemination was sudden, exceedingly vigorous, and produced 5–12 large, often multi-lobular, viable tumors with well-organized blood supplies at multiple sites (Supplementary Figure S2). Cells derived from individual metastatic sites on each animal were discretely characterized by karyotyping, whole genome Array-CGH analysis [21], recognized their cancer stem cell (CSC) physiognomies and stemness maintenance plasticity [22] and, defined their miRNA blue print and associated translational expression of tumor progression-related proteins [20]. Further, we utilized metastatic site-derived aggressive cells (MSDACs) and illustrated the tumorigenic capacity and reproducibility of the high-risk aggressive disease model (Supplementary Figure S2) [20, 21].

**Figure 2:**
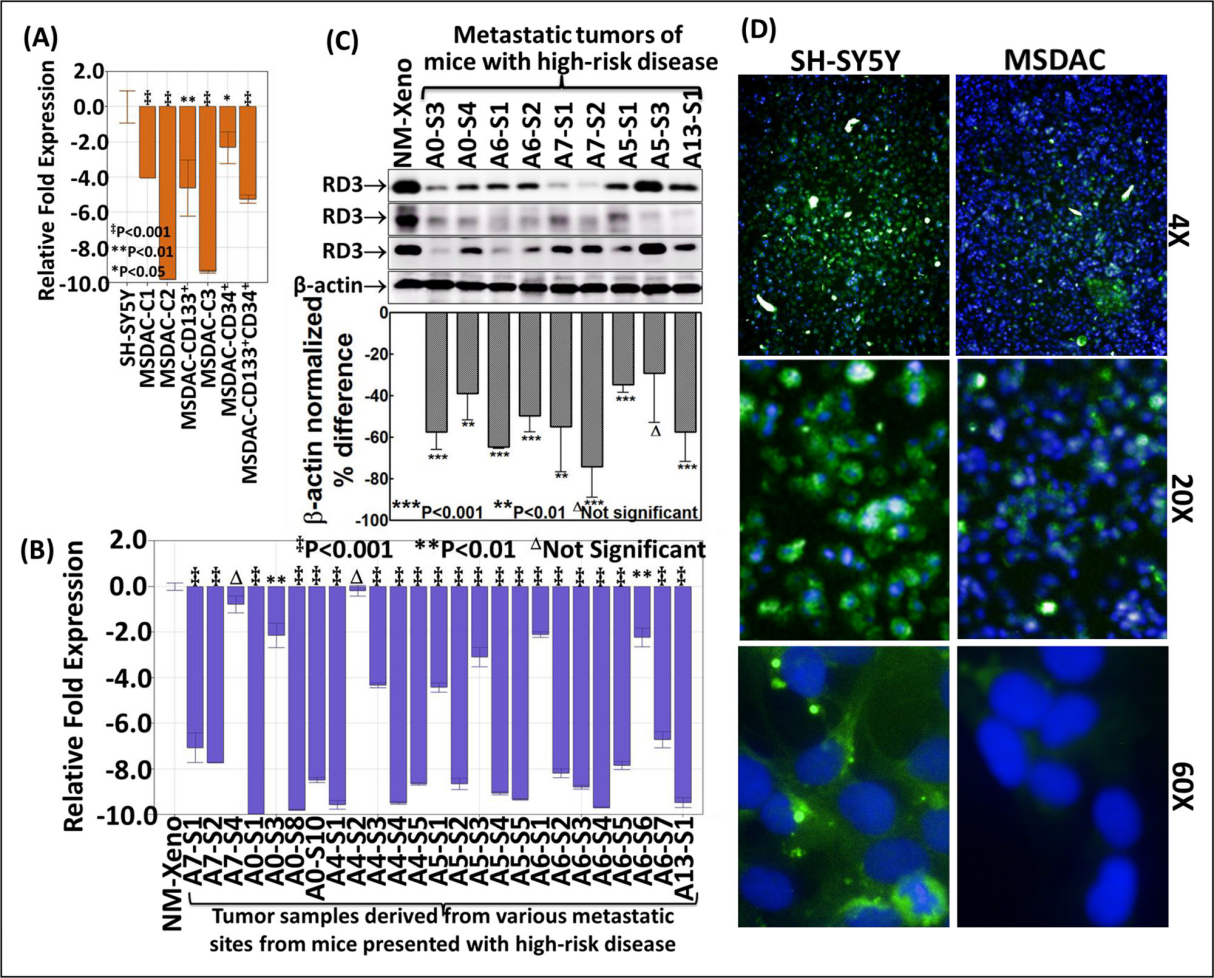
Transcriptional and translational loss of RD3 in high-risk aggressive neuroblastoma **A.** Histograms from QPCR analysis showing complete suppression of RD3-transcription in the clones of CD34^−^CD133^−^, CD133^+^CD34^−^, CD133^−^CD34^+^ or CD133^+^CD34^+^ MSDACs grown *ex vivo*. **B.** RD3-QPCR analysis in the manifold of tumor tissues from metastatic high-risk aggressive disease and non-metastatic xenografts. Compared to the non-metastatic primary xenografts, RD3-transcription was completely suppressed in every metastatic tumor. **C.** Representative immunoblots showing marked loss of RD3 in tumor tissues from metastatic sites. RD3 loss was examined with antibody from three different sources. Densitometry analyses was performed using Quantity One Image analysis software and are compared using GraphPad PRISM software **D.** High-content confocal imaging showing the cellular localization (60X) and the variation in RD3 levels in parental SH-SY5Y and MSDACs. RD3 is lost in MSDACs. Cellular localization and expression of RD3 in SH-SY5Y and MSDACs were examined using Operetta high content and quantitative confocal imaging using Alexa Fluor 488 fluorochrome conjugated anti-mouse secondary antibodies. The nucleus was counter-labeled with DAPI. Plates were analyzed in Operetta with atleast eight fields/well and three wells/clone with a minimum of 21-Z planes. Unstained controls were included for both cell lines.

To better characterize the loss of RD3 in high-risk neuroblastoma, ***first***, we investigated the transcriptional regulation of RD3 in MSDACs with or without CSC marker positivity. We maintained sequentially sorted CD133^−^CD34^−^, CD133^+^CD34^−^, CD133^−^CD34^+^ or CD133^+^CD34^+^ MSDACs (Supplementary Figure S3) *ex vivo* and analyzed for alterations in RD3 gene expression. QPCR revealed a complete (*P* < 0.001) decrease in RD3 mRNA levels in five different MSDAC clones examined compared with the parental SH-SY5Y cells (Figure 2A and Supplementary Figure S4A). We observed a significant decrease in the sorted NB CSCs, signifying the importance of ongoing acquisition of molecular alterations and associated tumor progression. However, as these MSDACs were grown *ex vivo* under controlled conditions, ***next***, we explored alterations in RD3 transcription in the tumors directly harvested from multiple metastatic sites from different animals. We compared the results with those from non-metastatic xenograft tissues. RD3 mRNA levels were significantly lower across the metastatic tumors investigated than in the xenograft tissues (Figure 2B). Though we observed inter-tumor variations in the mRNA expression levels of RD3, transcriptional loss of RD3 in metastasized tumors generally followed a consistent decrease over the manifold of non-metastatic primary xenograft controls (Supplementary Figure S4A).

### RD3 protein loss in mouse model of aggressive neuroblastoma

To examine RD3 loss using our unique mouse model of aggressive neuroblastoma, ***first***, we investigated differences in RD3 protein expression between the parental human SH-SY5Y cells and the clones of MSDACs. Compared to SH-SY5Y cells, immunoblotting analysis revealed a significant loss of RD3 in MSDAC clones examined (Supplementary Figure S4B). However, as discussed above, *ex vivo* culture and maintenance under controlled conditions may result in equivocal outcomes ***next***, we examined the alterations in the expression of RD3 protein in primary xenografts from animals with no metastasis and in the manifold of metastasized tumors from animals with aggressive disease. Immunoblotting revealed complete suppression of RD3 in metastatic tumors compared with non-metastatic xenograft controls (Figure 2C). This RD3 loss was consistent across multiple sites and animals. Quantity-One band densitometry analysis showed significant RD3 reduction in metastasized tumors compared to the non-metastasized primary xenografts. In addition, though we observed inter-tumor variations in the RD3 protein expression levels, comparison between the panel of xenografts to the metastasized tumors revealed a consistent loss of RD3 in metastasized tumors (Supplementary Figure S4B). ***Next***, high-content quantitative confocal imaging demonstrated RD3 loss in MSDACs cultured *ex vivo*, but not in the parental SH-SY5Y cell line (Figure 2D). High-magnification (20x and 60x) images validated the cellular localization of RD3 and clearly showed RD3 loss in MSDACs. These observations corroborated well with our immunoblotting results.

**Figure 3:**
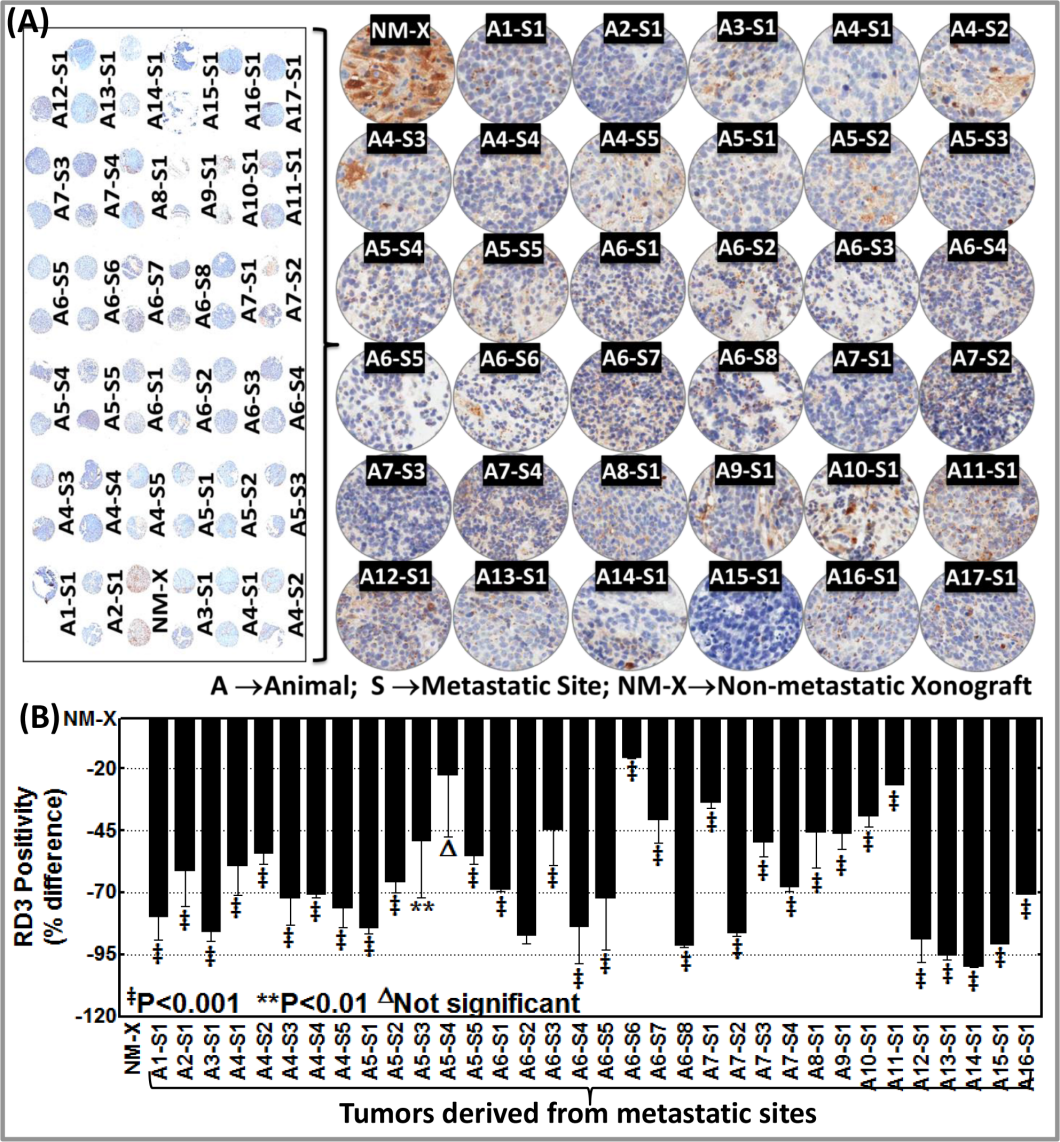
Customized tissue microarray analysis and automated RD3 IHC recognizes complete loss of RD3 in animal model of spontaneous and reproduced high-risk aggressive neuroblastoma **A.** Thumbnail and constructed images (20X) of tissue micro array (TMA) showing RD3 levels in non-metastatic primary xenograft (NM-X) and manifold of metastasized tumors from animals with spontaneous (developed from parental SH-SY5Y cells, A1–A12) and reproduced (developed from MSDACs, A13–A17) high-risk aggressive neuroblastoma. Tumor tissues from non-metastatic xenograft-bearing animals and from multiple metastatic sites from high-risk aggressive disease-bearing animals were printed in duplicate and examined for RD3 positivity and expression using automated IHC. **B.** Aperio image analysis coupled with GraphPad PRISM statistical analysis showing significant and consistent loss of RD3 across the manifold of metastasized tumors from animals with spontaneous and reproduced high-risk aggressive neuroblastoma when compared with non-metastatic primary xenograft. The slides were micro-digitally scanned using an Aperio Scanscope slide scanner and analyzed using integrated Spectrum software.

We then sought to define and typify the loss of RD3 in aggressive neuroblastoma. We used a custom-made TMA constructed with a manifold of tumors from the metastatic sites of several animals coupled with non-metastatic xenograft control. These were subjected to automated RD3 IHC, digitally scanned using Aperio and analyzed using Aperio Spectrum TMA image analysis. Consistent with our observations in normal mouse and human tissue controls (Figure 1), RD3 IHC staining revealed relatively strong positivity in non-metastatic xenograft (Figure 3A). We observed complete loss of RD3 in tumors metastasized to distant sites. This observation was consistent across the tumors from the same animal as well as tumors from different animals. We observed similar RD3 loss in the manifold of distant tumors reproduced from aggressive disease-bearing animals (Figure 3). Aperio image analysis demonstrated a complete and consistent loss of RD3 in the tumors of animals with aggressive disease (Figure 3B). Despite the inter-tumor and inter-animal variations in the loss of RD3 from the animals bearing spontaneous as well as reproduced high-risk aggressive neuroblastoma, comparison between the panel of primary xenografts to the panel of aggressive metastatic tumors demonstrated (i) consistent strong RD3 positivity in all non-metastatic primary xenografts and (ii) consistent and complete loss of RD3 in aggressive tumors (Supplementary Figure S4C).

### RD3 loss in clinical-high-risk aggressive neuroblastoma

**Figure 4:**
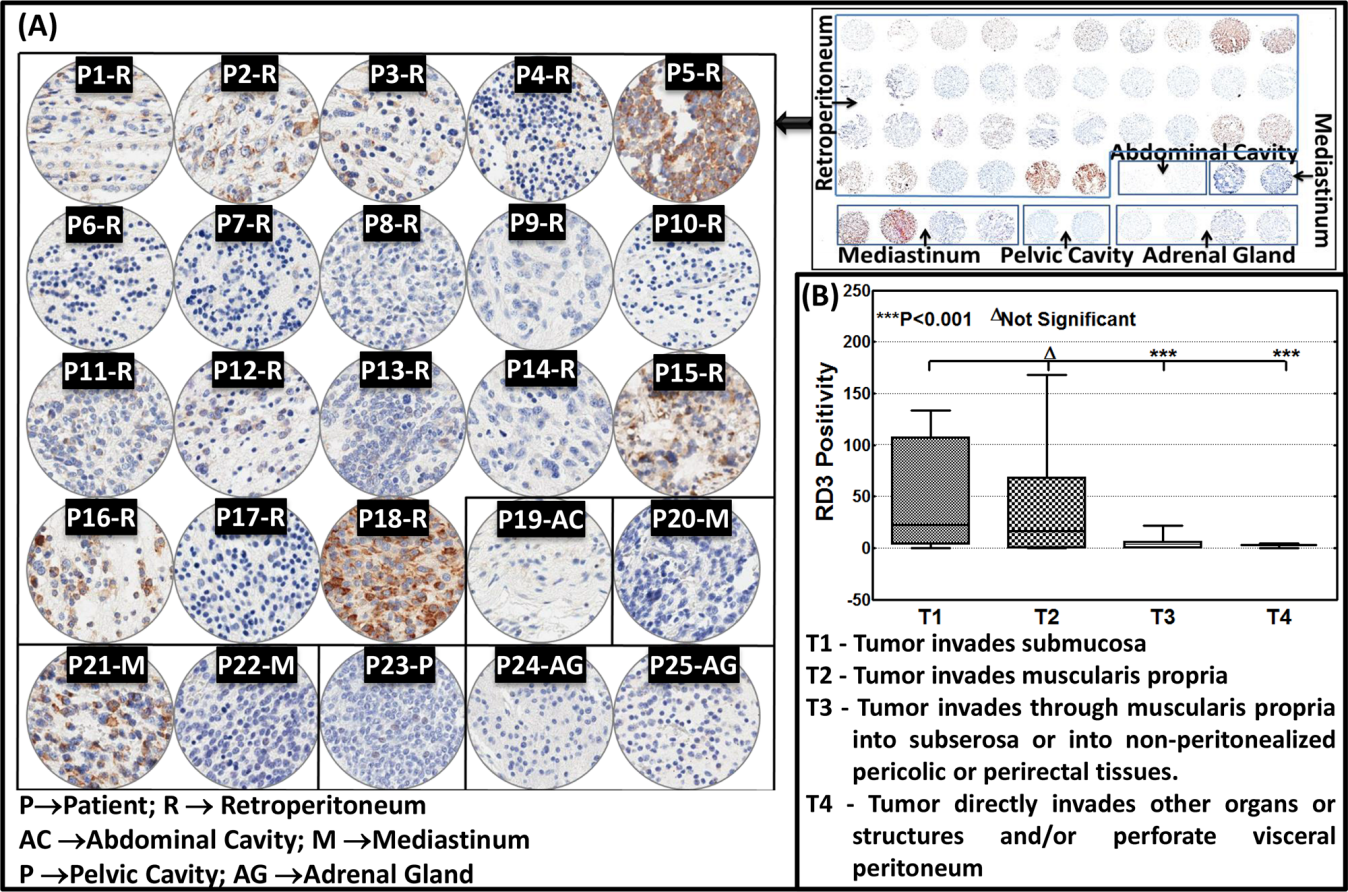
RD3 loss associates with the advanced tumor stage in neuroblastoma patients **A.** Thumbnail and constructed images (20X) of human neuroblastoma TMA showing RD3 levels in neuroblastoma samples (*n* = 25). TMA comprising human neuroblastoma tissues derived from the retroperitoneal, abdominal, and pelvic cavities, the mediastinum, and the adrenal glands subjected to automated RD3-IHC analysis revealed a significant correlation of the RD3 positivity with its expression decreased per increased tumor invasive potential, with complete loss in highly invasive T4 tumors to the TNM stages. TNM classification includes: Tumor invades submucosa (T1, *n = 5*); Tumor invades muscularis propria (T2, *n = 8*); Tumor invades through muscularis propria into subserosa or into non-peritonealized pericolic or perirectal tissues (T3, *n = 10*), and; Tumor directly invades other organs or structures and/or perforates visceral peritoneum (T4, *n = 2*). **B.** Aperio TMA image analysis of RD3 positivity identifies significant correlation of RD3 expression with TNM disease stage. Group-wise comparisons were performed with one-way ANOVA using GraphPad Prism.

To further substantiate RD3 loss in high-risk neuroblastoma in clinical settings, we utilized commercially available human neuroblastoma TMA. The tissues are derived from sites including the retroperitoneal, abdominal, and pelvic cavities, the mediastinum, and the adrenal glands. RD3-IHC analysis revealed a significant distinction in RD3 staining between patient samples (Figure 4). Consistent with the human control tissues (Figure 1B), RD3 positive staining was localized in the perinuclear, cytoplasmic, and nuclear regions (Figure 4A). Correlating the RD3 positivity to the TNM stages clearly identified the TNM stage-associated loss of RD3 (Figure 4B). TNM classification includes: Tumor invades submucosa (T1, *n = 5*); Tumor invades muscularis propria (T2, *n = 8*); Tumor invades through muscularis propria into subserosa or into non-peritonealized pericolic or perirectal tissues (T3, *n = 10*), and; Tumor directly invades other organs or structures and/or perforates visceral peritoneum (T4, *n = 2*). RD3 positivity directly correlated with T1, while its expression decreased per increased tumor invasive potential, with complete loss in highly invasive T4 tumors (Figure 4B).

### RD3 loss dictates tumor cell migration and invasion

**Figure 5:**
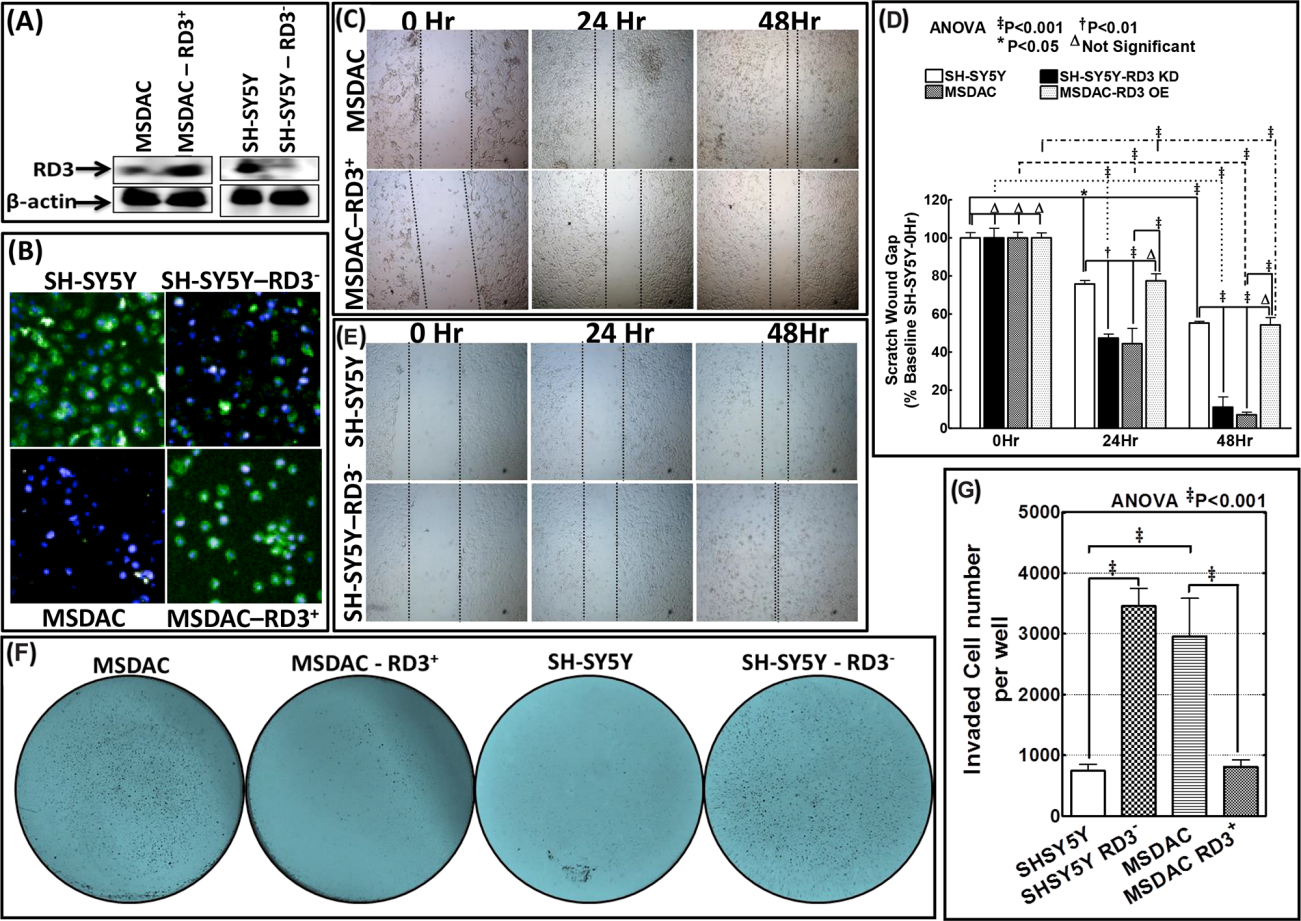
RD3 regulates tumor cell migration and invasion **A.** Representative immunoblots showing the re-expression of RD3 in MSDACs and silencing of RD3 in SH-SY5Y cells. Expression of GFP-tagged RD3 (Origene) was carried out by using TurboFectin 8.0 and RD3 silencing with shRNA (MISSION^®^ shRNA, Sigma-Aldrich) following standard protocols. **B.** Representative microphotographs acquired from Operetta high-content confocal immunofluorescence imaging validate RD3 silencing in SH-SY5Y cells and RD3 re-expression in MSDACs. **C.** Scratch-wound-assay showing the cell-migration patterns of MSDACs and RD3-re-expressed MSDACs under proliferation controlled conditions at 0, 24 and 48 h after wound initiation. MSDACs exhibits robust cell migrations with significant wound closure after 48 h, while re-expression of RD3 in MSDACs significantly inhibited their migration. **D.** Histograms of scratch wound gap measurements (mean and SD) showing the cell migration patterns of MSDACs with and without RD3 re-expression and parental SH-SY5Y cells with and without RD3 silencing examined at 0, 24 and 48 h after wound initiation. Group-wise comparisons were examined by two-way ANOVA with Bonferroni's post-hoc test made using GraphPad PRISM software and a *P* value of < 0.05 is considered significant. **E.** Scratch-wound-assay showing the cell-migration patterns of SH-SY5Y cells and RD3-silenced SH-SY5Y cells under proliferation controlled conditions at 0, 24 and 48 h after wound initiation. SH-SY5Y cells exhibited only base-line migrations after 48 h, while silencing RD3 in SH-SY5Y cells consistently increased their migration with significant wound closure. **F.** Representative microphotographs of matrigel invasion assay showing robust invasion of MSDACs, completely alleviated invasion in RD3-re-expressed MSDACs and profound increase in invasive potential of RD3-silenced SH-SY5Y cells. Invasion assays are performed using BD Matrigel invasion assay following standard protocols. **G.** Histograms of matrigel invaded cells (mean and SD) showing complete inhibition of MSDACs' invasion potential with RD3 re-expression and significant increase in the invasiveness of RD3-silenced SH-SY5Y cells. Quantification of invaded cells was performed using Image Quant colony count analysis software and the group-wise comparisons were examined by ANOVA with Bonferroni's post-hoc corrections using GraphPad PRISM software. A *P* value of < 0.05 is considered significant.

To define that the loss of RD3 drives the metastatic potential of NB cells and consequent metastasis, we examined its influence in tumor cell migration and invasion *in vitro* and in the instigation of aggressive disease with metastasis *in vivo*. For this, we adopted four different approaches: (i) re-expressed RD3 in aggressive RD3 lost MSDACs (Figure 5A *left panel* & 5B *lower panel*), (ii) muted RD3 in parental RD3 expressing SH-SY5Y cells (Figure 5A *right panel* & 5B *upper panel*) and analyzed for alterations in cell migration and invasion *in vitro*, (iii) stably re-expressed RD3 in MSDACs and (iv) stably silenced RD3 in parental SH-SY5Y cells and examined their sphere formation capacity *in vitro* as well as their tumorigenesis and metastatic potential *in vivo*. Under proliferation controlled conditions, scratch-wound assay demonstrated a profound (*P* < 0.001) migration of MSDACs as early as 24 h and exhibited a near-complete (*P* < 0.001) closure of wound after 48 h (Figure 5C *top panel* and Figure 5D). On the other hand, re-expression of RD3 in these aggressive MSDACs resulted in the inhibition of their migration potential (Figure 5C *bottom panel*). Evidently, compared to MSDACs migration potential, RD3 re-expression resulted in a significant (*P* < 0.001) inhibition of cellular migration both after 24 h and 48 h (Figure 5D). These results mimic observed cell migration patterns of parental SH-SY5Y cells that have high-levels of constitutive RD3 (Figure 5E *top panel* and Figure 5D). Conversely, muting RD3 in SH-SY5Y cells significantly increased their migration potential as early as 24 h (*P* < 0.01) and 48 h (*P* < 0.001) recognizing the defined role of RD3 silencing in cellular migration (Figure 5E *bottom panel* and Figure 5D).

***Next***, matrigel invasion assay recognized the robust metastatic potential of MSDACs with significant number of cells invading (Figure 5F). Compared to human SH-SY5Y cell, this increase in invasiveness of MSDACs remained statistically significant (Figure 5G). Conversely re-expressing RD3 significantly (*P* < 0.001) delimited MSDACs' invasion potential (Figure 5F & 5G) and levels with SH-SY5Y invasiveness capacity. On the other hand, silencing RD3 in SH-SY5Y cells profoundly (*P* < 0.001) enhances their invasive potential (Figure 5F) and parallels with the MSDACs invasiveness (Figure 5G).

**Figure 6:**
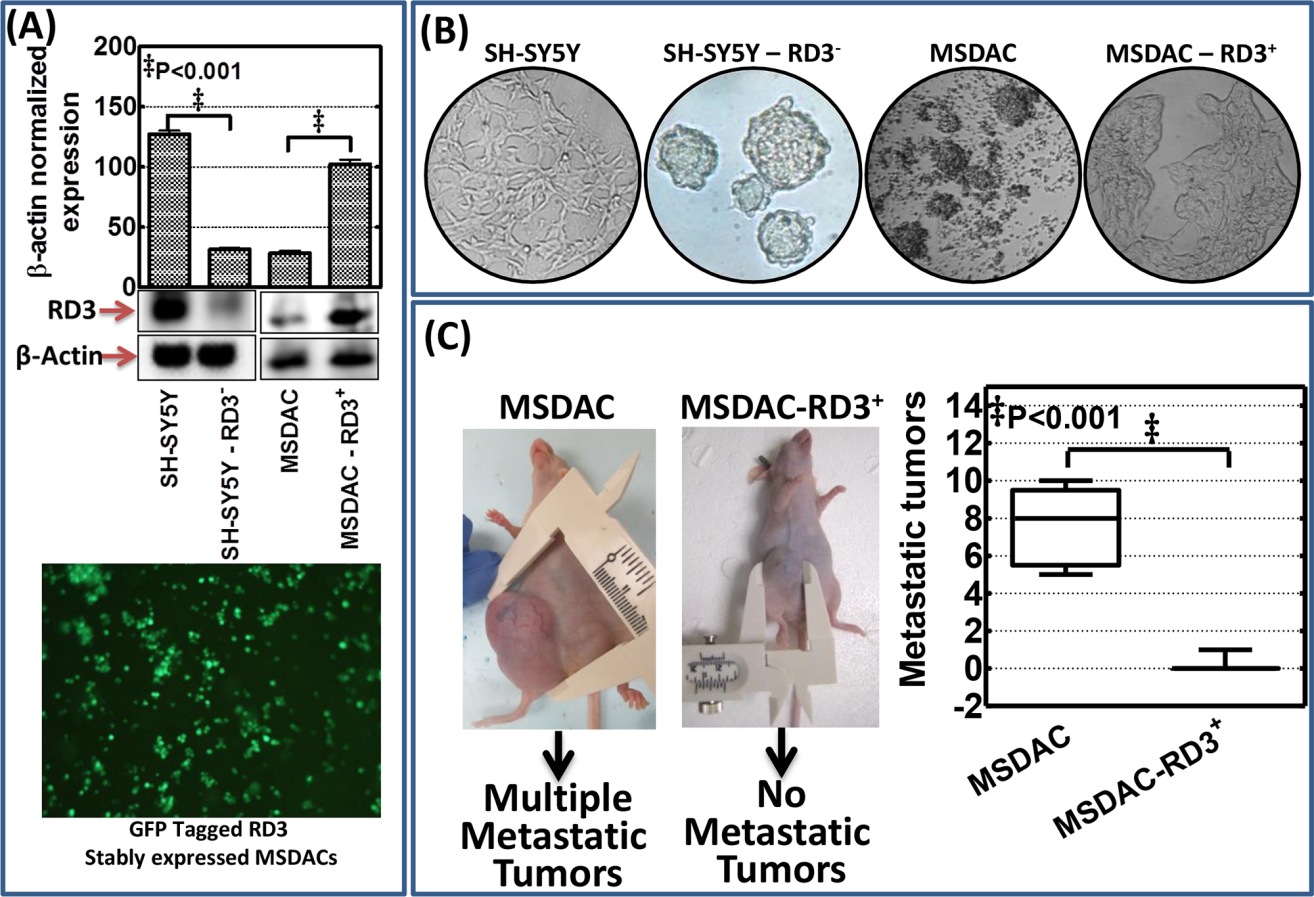
RD3 regulates metastatic potential of neuroblastoma cells **A.** Representative immunoblots showing RD3 knocked down in human SH-SY5Y cells stably transfected with RD3 shRNA (MISSION^®^ shRNA, Sigma-Aldrich) with puromycin mammalian selection and re-expression of RD3 in MSDACs that were stably transfected with RD3 (GFP-tagged - Human retinal degeneration 3, transcript variant 1, Origene Technologies) with neomycin mammalian selection. The stable transfection was carried out using either TurboFectin 8.0 reagent (Origene) or Neon electroporation transfection system (Life Technologies). Representative photomicrograph showing cells stably transfected with GFP tagged RD3 construct showing the expression of GFP. **B.** Representative phase contrast photomicrographs showing tumorosphere forming capabilities of SH-SY5Y (vc), RD3 stably silenced SH-SY5Y cells, MSDACs (vc) and RD3 stably re-expressed MSDACs maintained in serum free stem cell culture conditions. **C.** Schematic representation and representative mouse images showing relative tumorigenic capacity and aggressive disease formation of RD3 stably expressing MSDACs. RD3 stably expressing MSDACs resulted in the development of relatively small xenograft (~250 mm^3^) without metastatic tumors compared with the large xenografts with multiple metastasis in mice that received MSDACs (vc). Vertical Box and Whiskers plot showing mean number of metastatic tumors observed in animals that received MSDACs and RD3 re-expressed MSDACs.

***Next***, Modified sphere limiting dilution analysis with DiI staining and observed in real-time for a period of 18 h recognized the role of RD3 in tumorosphere formation (Supplementary Video S1). SH-SY5Y cells exhibited monolayer cell spreading without any organized tumorosphere formations in serum free stem cell medium, SF-SCM (Supplementary Video S1 *Top left panel*). Conversely, silencing RD3 in SH-SY5Y cells resulted in the formation of organized tumorospheres and defied any monolayer cell spreading (Supplementary Video S1 *Bottom left and middle panel*). On the other hand, MSDACs exhibited a robust and organized tumorosphere formation without any monolayer cell spreading (Supplementary Video S1 *Top right and middle panel*). Interestingly, re-expressing RD3 in MSDACs completely abrogated organized tumorosphere formation (Supplementary Video S1 *Bottom left panel*). However, it is pertinent to note that we did not see any monolayer cell spreading in these RD3 re-expressed MSDACs. Substantiating further, under serum free stem cell culture conditions while MSDACs formed organized tumorospheres, stably re-expressing RD3 (Figure 6A) completely level-down this tumorosphere forming capacity in these aggressive MSDACs (Figure 6B). On the other hand, muting RD3 in SH-SY5Y cells (Figure 6A) resulted in the formation of organized tumorospheres consistently across generations (Figure 6B). More importantly, stably re-expressing RD3 in MSDACs significantly reduced their *in vivo* metastatic potential. Administering the RD3 re-expressed MSDACs resulted in relatively smaller xenografts without any metastasis (Figure 6C) with an exception of single incidence where we observed a small retroperitoneal metastasis. Together, these results demonstrate that the loss of RD3 arbitrate aggressive metastatic neuroblastoma.

### RD3 loss affects clinical outcomes

We examined the correlation of RD3 expression with overall survival, relapse-free survival, disease stage, prognosis, and patient status. We used the R2: microarray analysis and visualization platform (http://r2.amc.nl) by Dr. Jan Koster at the Department of Oncogenomics in the Academic Medical Center (AMC), Amsterdam, Netherlands. This web-based application correlates a select gene expression profile with clinical outcomes for various cohorts of patient samples that are submitted by individual investigators. In a cohort of 88 neuroblastoma patients, low RD3 expression was inversely correlated with overall patient survival (Supplementary Figure S5A). Further, this negative correlation of RD3 loss was magnified when computed for relapse-free survival (Supplementary Figure S5B). This cohort showed clear loss of RD3 in patients with high-risk 4S disease, as opposed to favorable stage 1 disease (Supplementary Figure S5C). Another cohort of 47 NB patients also showed significantly less (*P* < 0.03) RD3 expression in stage 4 than stage 1 disease (Supplementary Figure S5D). Additional studies with a cohort of 30 NB patients validated the complete loss of RD3 in high-risk aggressive metastatic stage 4 neuroblastoma (Supplementary Figure S5E). Interestingly, a study with a cohort of 64 neuroblastoma patients demonstrated a complete loss of RD3 as the tumor progressed (Supplementary Figure S5F). RD3 loss correlated highly with prognosis and patient status. These data show that there is a significant loss of RD3 as the disease progresses. RD3 level is substantially correlated with overall and relapse-free survival.

## DISCUSSION

Clinical outcome remains poor in patients with high-risk aggressive neuroblastoma, despite intensified multimodal treatment. A considerable proportion of high-risk patients experience frequent disease relapses [9, 10], with low rates of five-year OS (<10%) and almost no long-term survival [13, 15]. Considering that nearly half of neuroblastoma patients possess favorable disease with spontaneous regression/maturation, the progression, relapse, and subsequent death in patients with high-risk disease may reflect the ongoing acquisition of genetic manipulations and/or pro-oncogenic adaptations in the undifferentiated tumorigenic neural crest cells that could switch the disease risk status. In this study, we found that RD3, a retinal degeneration protein, is completely lost in patients with high-risk invasive disease, but not in patients with less invasive tumors. Decreased RD3 expression was directly associated with the increase in progressive phenotype. Using a clinically relevant animal model of high-risk human neuroblastoma, we found that RD3 transcriptional machinery is regulated in high-risk disease. Further, our established animal model reflected RD3 loss in aggressive metastatic tumors. Nearly all metastatic tumors showed complete RD3 loss, while the non-metastatic tumors did not. More importantly, the MSDACs derived from tumors that lack RD3 instantaneously prompt the development of high-risk aggressive metastatic disease. Evidently, gene manipulation studies demonstrated that loss of RD3 drives neuroblastoma cell migrations as well as heightened metastatic potential. To our knowledge, this the first report of RD3 loss in aggressive neuroblastoma, or in any other tumor system.

RD3 has been extensively studied in the context of eye degeneration. It has been shown that truncation mutations in this gene are responsible for photoreceptor degeneration and inflict early onset of vision loss in patients with Leber Congenital amaurosis 12 [16, 19, 23–25]. Although RD3 has been shown to be associated with the leukemia gene product PML [17], the crucial role of RD3 in cancer biology has been overlooked. To better underscore the functional role of RD3 in neuroblastoma progression, it is necessary to determine its cellular localization and expression in healthy tissues as well as organ/tissue-specific variations. In this study, we found that RD3 is ubiquitously expressed in the human colon, brain, liver, kidney, pancreas, and spleen. We also determined that RD3 was localized in the perinuclear, cytoplasmic, and nuclear regions. In addition, with mouse macroarray RD3 IHC, we found similar cellular localization and ubiquitous expression in the mouse liver, kidney, brain, and spleen.

We also assessed data from multiple cohorts of neuroblastoma patients to examine the correlation of RD3 expression with clinical outcomes, disease stage, prognosis, and patient status (http://r2.amc.nl). Data analysis identified a strong correlation between RD3 loss and poor OS. There was significantly less relapse-free survival in children with low RD3 expression, regardless of age or gender. In every cohort of neuroblastoma patients, we found that RD3 loss significantly correlates with disease progression, with maximal loss in stage 4/4S. This clinical data correlation, alongside our human TMA data correlations and results from *in vivo* experiments, directly demonstrates the definitive impact of RD3 loss in neuroblastoma progression. Coupled with the clinical evidence, our *in vivo* demonstration of aggressive disease formation in cells lacking RD3 shows the crucial role of RD3 in this setting.

Given the ongoing acquisition of molecular/genetic alterations in high-risk aggressive neuroblastoma, we strongly believe that the results of this study provide a critical piece of the puzzle regarding the transition from favorable/responsive disease to high-risk disease. Human TMA data from the laboratory, clinical correlation data from manifold of neuroblastoma patient cohorts as well as the *in vivo/ex vivo* laboratory investigations unidirectionally dictates that RD3 loss mediates neuroblastoma progression. However, further studies are warranted to understand the direct role of RD3 in the exertion of the switch from favorable to unfavorable disease with appropriate clinically translatable transgenic approaches. Further, the upstream functional molecular orchestration and/or genetic responses that drive RD3 loss must be determined. In this direction, *in vitro/in vivo* gene manipulation studies coupled with knock-in/knock-out double transgenic approaches are currently underway in our laboratory to pinpoint the role of RD3 loss in tumor invasion, metastasis, and neuroblastoma progression.

RD3 loss is correlated with high-risk aggressive neuroblastoma. In this study, we found that RD3 is significantly lost in human neuroblastoma tissues. The loss of RD3 is associated with progressive disease. In the animal model of human neuroblastoma, we found complete RD3 loss in a manifold of tumors from metastatic sites. Aggressive cells that were derived from metastatic tumors and lacked RD3 instantaneously initiated and endorsed high-risk aggressive disease with multiple metastases. Localization and expression studies revealed a ubiquitous expression of RD3 in healthy human and mouse tissues. Gene manipulation approaches recognized that RD3 loss mediates tumor cell migration and metastatic potential in neuroblastoma cells. Correlation analysis from multiple cohorts of neuroblastoma identified a strong association between RD3 loss and clinical outcomes, advanced disease, and poor OS and relapse-free survival. This study recognizes and defines RD3 loss and associated clinical outcomes in high-risk aggressive metastatic neuroblastoma.

## MATERIALS AND METHODS

### Cell culture

The human neuroblastoma (SH-SY5Y) cells were obtained from ATCC (Manassas, VA) and were cultured and maintained as described earlier [26]. SH-SY5Y cells are a sub-line (SK-N-SH → SH-SY → SH-SY5 → SH-SY5Y) of the parental cell line SK-N-SH, which is established from a metastatic bone tumor and is composed of neuroblast-like (N-type) floating and substrate-adherent (S-type) epithelial-like cells.

### Development of reproducible non-metastatic xenografts and metastatic aggressive disease mouse models

All animal experiments conformed to American Physiological Society standards for animal care and were carried out in accordance with guidelines from the National Research Council. Protocols were approved by our IACUC. Neuroblastoma xenograft and/or aggressive metastatic disease development was discussed in our earlier studies [20–22]. Tumor growth, regression, and dissemination to distant sites were examined with tumor volume measurements and non-invasive fluorescent imaging (IntegriSense 750, Perkin Elmer, Inc.). Animals were euthanized by CO_2_ asphyxiation. Single-cell suspensions and cell derivations from the harvested tumors from metastatic sites and non-metastatic xenografts were performed as discussed in our earlier studies [20–22]. Derived single cell suspensions were grown *ex vivo* in stem cell medium (DMEM:F12 with 1% N2 Supplement, 2% B27 Supplement, 20 ng/ml hPDGF, 100 ng/ml EGF, and 1% antibiotic-antimycotic). Cells were sorted thrice by FACS influx cell sorter, carefully defining their phenotypes and physiognomies by employing proper control. Briefly, for every cell population sorted, we adopted a sequential set of exclusion and inclusion criteria to isolate human CD133^+^CD34^+^ NB-CSCs from the metastatic sites. Cells were divided into six samples: unstained, CD133^+^PE, CD34^+^APC, mouse CD31-biotin + mouse lineage-biotin + mouse H-2Kd-biotin, IgG2bκ-APC + IgG2aκ-PE, and CD34^+^APC + CD133^+^PE + mouse CD31-biotin + mouse lineage-biotin + mouse H-2Kd-biotin. CSCs were screened based on CD133^+^CD34^+^ subtypes. To reproduce high-risk disease, animals were injected with isolated and well-characterized clones of aggressive cells derived from individual metastatic sites and were then observed for the metastatic tumors.

### QPCR

We used real-time QPCR to analyze the transcriptional regulation of RD3 in metastatic tumor-derived aggressive cells, with or without CD133 and/or CD34 positivity, grown *ex vivo*, and in tissues from individual metastatic tumors from multiple mice that presented with high-risk aggressive disease as described earlier [26] [27].

### Immunoblotting

Total protein extraction and immunoblotting were performed as described in our earlier studies [26, 28]. For this study, the protein transferred membranes were incubated with either mouse monoclonal (Santa Cruz Biotechnology, Inc., Dallas, TX) or rabbit polyclonal and mouse monoclonal anti-human RD3 [16] and were developed with the appropriate anti-mouse/anti-rabbit (BioRad Laboratories, Hercules, CA) secondary antibody. Blots were stripped and reblotted with rabbit polyclonal anti-β-actin antibody (Gentex Inc., Irvine, CA) to determine equal loading of the samples.

### Confocal immunocytofluorescence

We examined the cellular localization and expression levels of RD3 in parental SH-SY5Y and MSDACs using Operetta (Perkin Elmer) high content and quantitative confocal imaging. Paraformaldehyde fixed SH-SY5Y and MSDACs were permeabilized (0.25% Triton X-100), blocked (1% BSA in PBS), and labelled with mouse monoclonal anti-RD3 (1: 200, Santa Cruz). Then, they were tagged with Alexa Fluor 488 fluorochrome conjugated anti-mouse secondary antibodies (Abcam). The nucleus was counter-labeled with DAPI. After washing, the plates were analyzed in Operetta with at least eight fields/well and three wells/clone, with a minimum of 21 Z planes. Unstained controls were included for both cell lines. Columbus software (Perkin Elmer) was used for quantitative image analysis.

### Tumorosphere formation capacity

We examined the influence of RD3 in the regulation of tumorosphere formation capacity using limiting dilution tumorosphere formation assay in MSDACs with and without RD3 re-expression and in SH-SY5Y cells with and without RD3 silencing. For this, we used Operetta (Perkin Elmer, Inc., Waltham, MA) high-content real-time fluorescent imaging to examine serially diluted cells plated in 96-well culture plates and stained with DiI Stain, an orange-red-fluorescent dye that is a long-term tracer for neuronal cells [1,1′-Dioctadecyl-3,3,3′,3′-Tetramethylindocarbocyanine Perchlorate (‘DiI’; DiIC18(3)), Life Technologies, Grand Island, NY]. Images were acquired for every 20 min for period of 18 h. Sequential images were reconstructed in Harmony (Perkin Elmer) to obtain a time-lapse video.

### Plasmid preparation and DNA transfection

Expression of RD3 (GFP-tagged - Human retinal degeneration 3, transcript variant 1, Origene) was carried out by using TurboFectin 8.0 reagent (Origene). RD3 silencing was achieved using shRNA (MISSION^®^ shRNA, Sigma-Aldrich).

### Scratch-wound assay

The alterations in cell migration in response to the re-expression of RD3 in MSDACs or silencing RD3 in parental SH-SY5Y cells were examined using scratch-would assay as described earlier [29]. Mitomycin C (10 μg/ml, Sigma) was used to arrest cell proliferation. All experiments were repeated at least five times in each group.

### Matrigel invasion assay

For invasion assays, 1 × 10^5^ cells were plated in the top chamber with Matrigel-coated membrane (24-well insert; pore size, 8 μm; BD Biosciences). Cells were plated in medium without serum and medium supplemented with serum was used as a chemo attractant in the lower chamber. The cells were incubated for 24 h and cells that did not migrate or invade through the pores were removed by a cotton swab. Cells on the lower surface of the membrane were fixed with 3:1 methanol: acetic acid and stained using 0.1% Crystal violet to visualize the Invaded cells.

### Tissue macroarray/microarray construction and, quantitative immunohistochemistry

All mouse tissue macroarray and microarray construction and IHC staining were performed in the Stephenson Cancer Center Cancer Tissue Pathology Core. The normal mouse tissue macroarray was constructed with mouse brain, kidney, liver, and spleen tissues. Since the published literature has only reported data on RD3 in the eye, we used parallel sections of mouse whole eyes as controls. For mouse neuroblastoma TMA, tumor tissues from non-metastatic xenograft-bearing animals and from multiple metastatic sites from high-risk aggressive disease-bearing animals were printed in duplicate. All H & E stained slides were reviewed for pathology. The slides were micro-digitally scanned using an Aperio Scanscope (Aperio Technologies, Inc.,) slide scanner and analyzed using integrated Spectrum software.

To better understand changes in RD3 expression in clinical subjects, we used a commercially available human neuroblastoma tissue array (Cat. No. MC-602, US Biomax, Inc., Rockville, MD). The 5 μm thick human TMA is equipped with duplicate 1.5 mm cores of neuroblastoma tissues from various sites including the retroperitoneum, mediastinum, abdominal and pelvic cavities, and the adrenal glands of 25 patients. Further, the TMA is armed with clinical variables including sex, age, site/organ, diagnosis, and TNM grading. RD3 positivity for the cores was then correlated with TNM stages. Group-wise comparisons were performed with one-way ANOVA (GraphPad Prism).

## SUPPLEMENTARY FIGURES AND VIDEO


