# Supplementary material for: RD3 loss dictates high-risk aggressive neuroblastoma and poor clinical outcomes
Source: Oncotarget. 2015 Sep 8;6(34):36522–34. doi: 10.18632/oncotarget.5204 (PMC4742193; doi:10.18632/oncotarget.5204)
Supplement: Supplementary file 1 [file oncotarget-06-36522-s001.pdf]

SUPPLEMENTARY FIGURES AND VIDEO

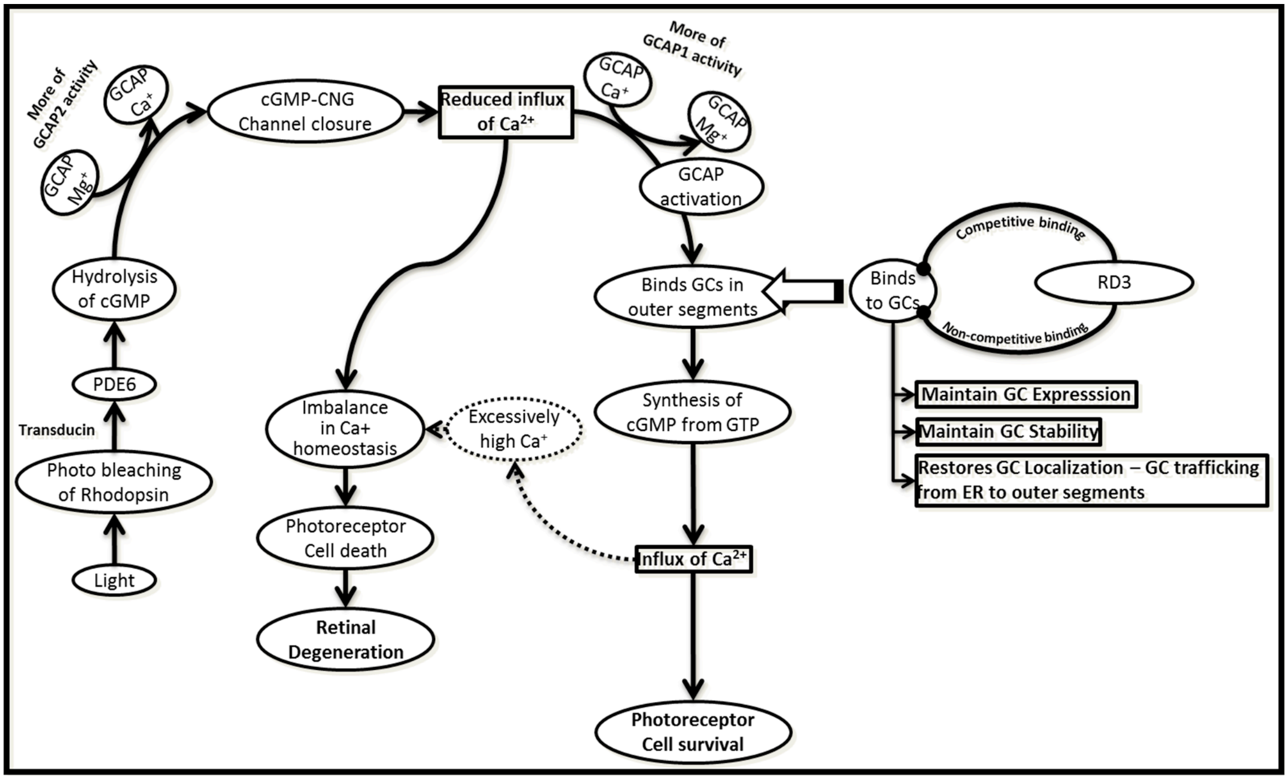

Supplementary Figure S1: Schematic representation of the role of Retinal Degeneration Protein RD3 in photoreceptor cell survival. Chart showing the mechanistic signal transduction flow through that affect photoreceptor cell survival and consequent retinal degeneration and, functional regulatory role of RD3 in this setting.

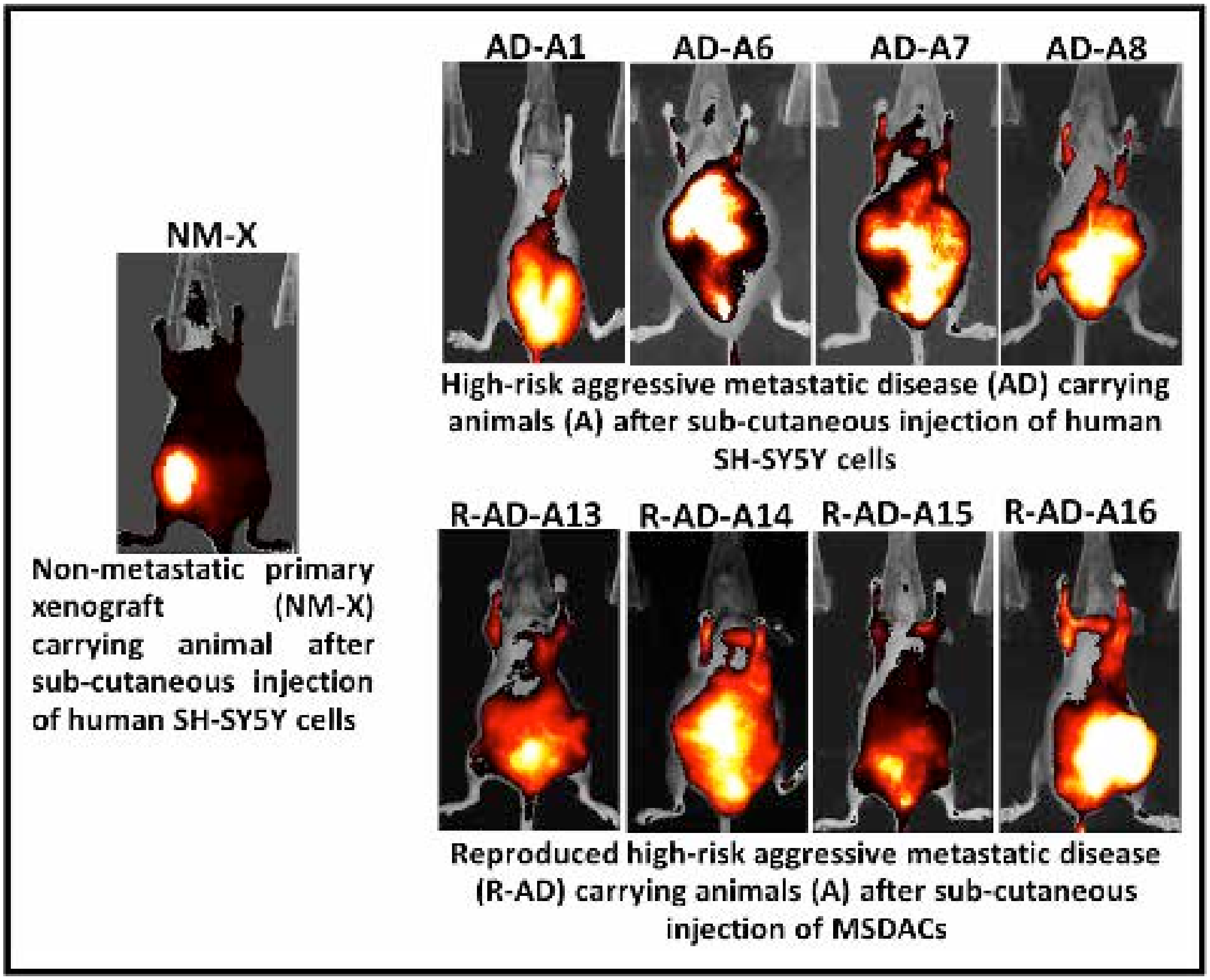

Supplementary Figure S2: *In vivo* mouse model of reproducible high-risk aggressive metastatic neuroblastoma. ***Left:*** *In vivo* fluorescent non-invasive tumor imaging showing neuroblastoma xenograft without any metastasis in animals that received sub-cutaneous injection of human SH-SY5Y cells in matrigel. ***Top Panel:*** *In vivo* fluorescent non-invasive tumor imaging showing the development of high-risk aggressive disease with tumor metastasis (in pelvic, retroperitoneal, abdominal and in chest cavities) in animals that received identical clones of SH-SY5Y cells under identical conditions. ***Bottom Panel:*** *In vivo* tumor imaging showing the reproducibility of high-risk aggressive disease with tumor metastasis (in pelvic, retroperitoneal, abdominal and in chest cavities) in animals that received metastatic site derived aggressive cells (MSDACs).

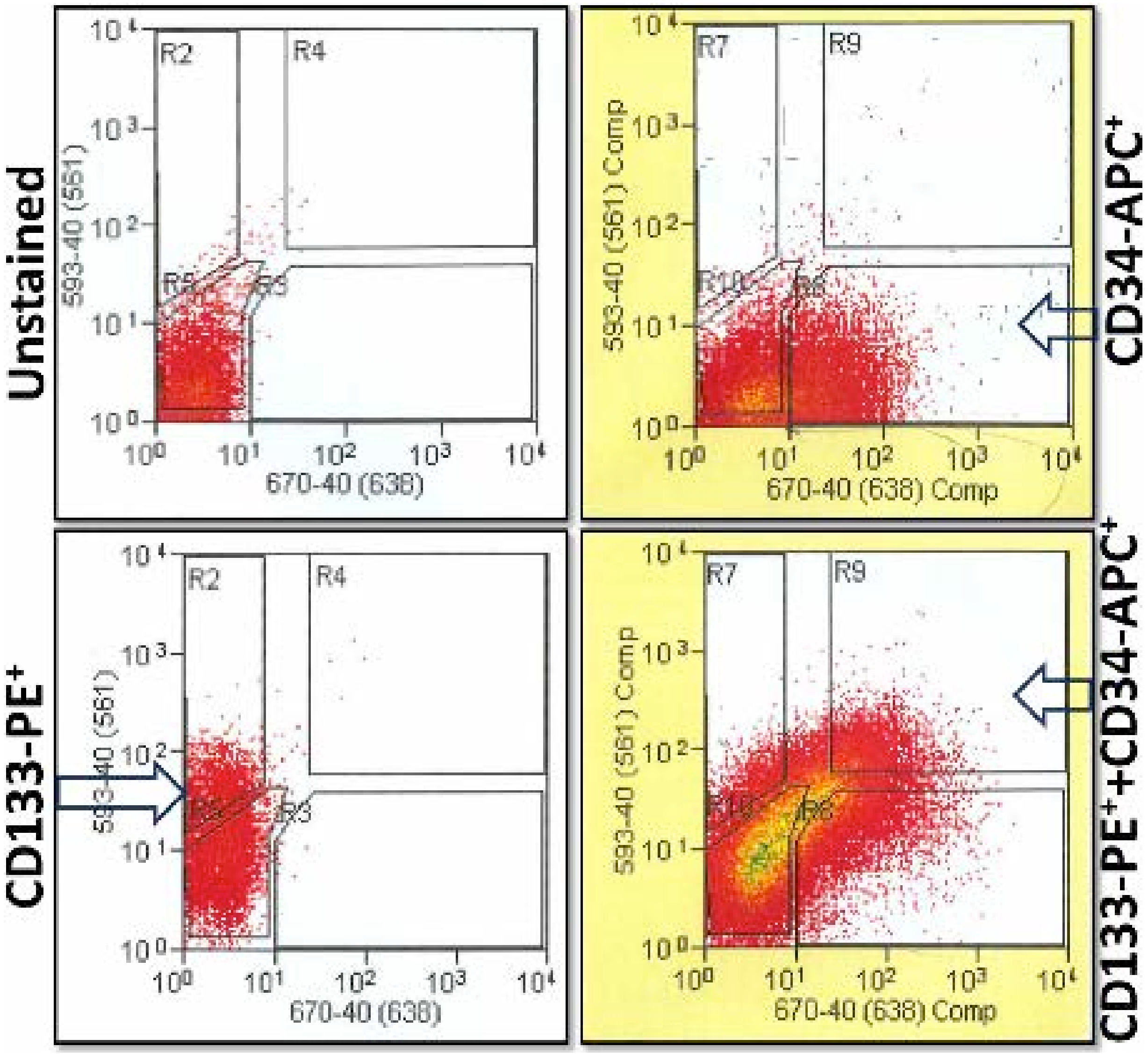

Supplementary Figure S3: Flow-cytometry cell sorting of CD133$^+$+CD34$^+$ human NB CSCs. Human CD133$^+$+CD34$^+$ neuroblastoma cells derived from non-metastatic xenografts or from the manifold of metastatic sites from different animals was sorted with the gate created based on the viable non-mouse derived cells (by excluding PI$^+$, mouse CD31$^+$, mouse lineage$^+$ or mouse H-2Kd$^+$), and the IgG2bk-APC (isotypic control for CD34-APC) and IgG2ak-PE (isotypic control for CD133-PE) antibodies.

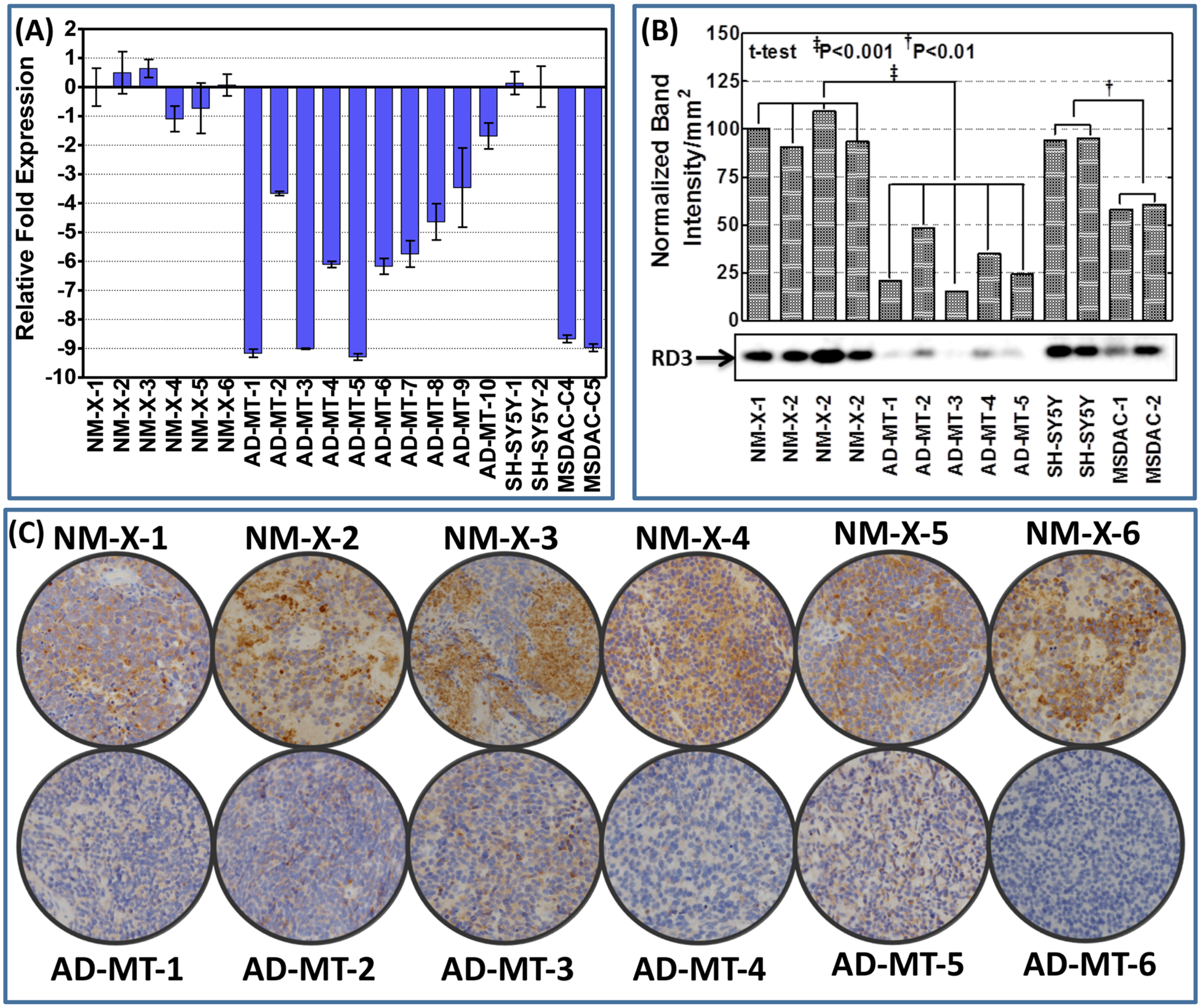

Supplementary Figure S4: Loss of RD3 in high-risk aggressive neuroblastoma. A. Histograms from QPCR analysis showing complete suppression of RD3-transcription in the manifold of tumor tissues from metastatic high-risk aggressive disease compared to the manifold of primary xenografts from the animals without aggressive disease and, in the clones of MSDACs cultured and maintained *ex vivo* compared to the clones of parental human SH-SY5Y cells. **B.** Representative immunoblots showing marked loss of RD3 in manifold of metastasized tumor tissues ($n = 5$) from aggressive disease bearing animals compared to the manifold of primary xenografts ($n = 4$) from animals that showed no aggressive disease. Densitometry analyses was performed using Quantity One Image analysis software and are compared using GraphPad PRISM software **C.** Representative photomicrographs (20X) showing RD3 localization and expression levels in a manifold of non-metastatic xenografts and, multifarious metastatic tumors. Automated RD3 IHC staining revealed strong and consistent positivity in non-metastatic xenografts and further demonstrated a complete loss of RD3 in metastasized tumors.

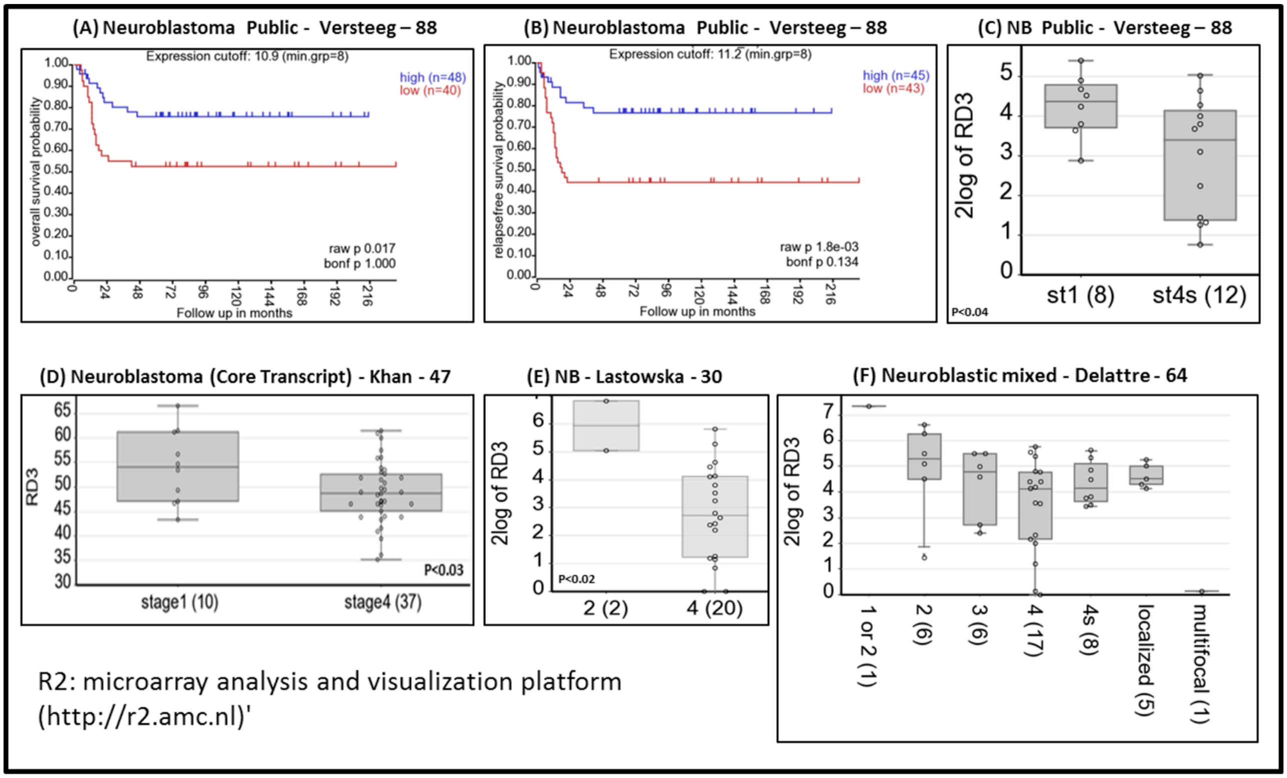

Supplementary Figure S5: RD3 loss associates with poor clinical outcomes. Correlation of RD3 expression with NB clinical outcomes in a cohort of 88 patients. **A.** Kaplan-Meier curve showing decreased OS in patients with low RD3 expression compared with high RD3 expression. **B.** Kaplan-Meier curve showing a pronounced decrease in relapse-free survival for patients with low RD3 expression. **C.** RD3 expression and disease progression: Box-whiskers plot with circles showing significant loss of RD3 in patients with high-risk 4S disease. **D.** Independent cohort of 47 patients showing significant decrease in RD3 expression between stage 1 and stage 4. **E.** Box-whiskers plot with circles showing complete loss of RD3 in high-risk aggressive metastatic stage 4 disease in a separate cohort of 30 patients. **F.** Graph showing stage-wise RD3 expression in a cohort of 64 patients. RD3 levels were high at stages 1 and 2. Levels decrease with disease progression and are significantly lower in multifocal disease.

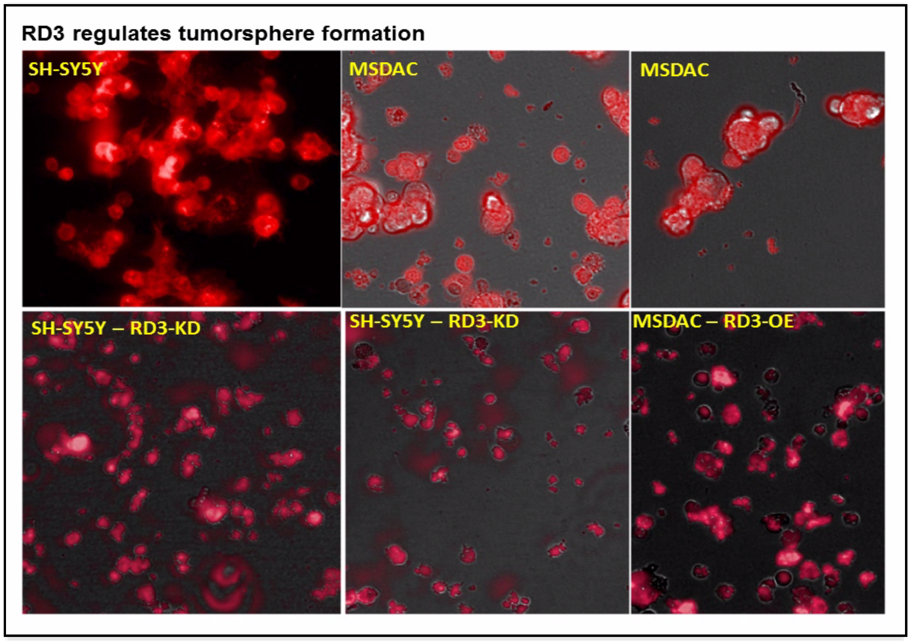

Supplementary Video S1: *RD3 regulates* tumorosphere formation capacity. Representative video clips from limiting dilution tumorosphere formation assay showing the influence of RD3 in the regulation of tumorosphere formation capacity. Serially diluted SH-SY5Y cells with and without RD3 silencing or MSDACs with and without RD3 re-expression plated in 96-well culture plates were stained with DiI, and were imaged in high-content real-time fluorescent imager, Operetta for every 20 min for period of 18 h. SH-SY5Y cells exhibited monolayer cell spreading without any organized tumorosphere formations (*Top left panel*). Silencing RD3 SH-SY5Y cells resulted in the formation of organized tumorospheres and defied any monolayer cell spreading (*Bottom left and middle panel*). MSDACs exhibited organized tumorosphere formation without any monolayer cell spreading (*Top right and middle panel*). Re-expressing RD3 in MSDACs completely abrogated organized tumorosphere formation (*Bottom left panel*).
